# Beat cues facilitate time estimation at longer intervals

**DOI:** 10.3389/fpsyg.2023.1130788

**Published:** 2023-09-29

**Authors:** Nathércia L. Torres, São Luís Castro, Susana Silva

**Affiliations:** Speech Laboratory, Faculty of Psychology and Education Sciences, University of Porto, Porto, Portugal

**Keywords:** beat cues, time estimation, duration-based system, beat-based system, perception

## Abstract

**Introduction:**

Time perception in humans can be relative (beat-based) or absolute (duration-based). Although the classic view in the field points to different neural substrates underlying beat-based vs. duration-based mechanisms, recent neuroimaging evidence provided support to a unified model wherein these two systems overlap. In line with this, previous research demonstrated that internalized beat cues benefit motor reproduction of longer intervals (> 5.5 s) by reducing underestimation, but little is known about this effect on pure perceptual tasks. The present study was designed to investigate whether and how interval estimation is modulated by available beat cues.

**Methods:**

To that end, we asked 155 participants to estimate auditory intervals ranging from 500 ms to 10 s, while manipulating the presence of cues before the interval, as well as the reinforcement of these cues by beat-related interference within the interval (vs. beat-unrelated and no interference).

**Results:**

Beat cues aided time estimation depending on interval duration: for intervals longer than 5 s, estimation was better in the cue than in the no-cue condition. Specifically, the levels of underestimation decreased in the presence of cues, indicating that beat cues had a facilitating effect on time perception very similar to the one observed previously for time production.

**Discussion:**

Interference had no effects, suggesting that this manipulation was not effective. Our findings are consistent with the idea of cooperation between beat- and duration-based systems and suggest that this cooperation is quite similar across production and perception.

## Introduction

Dealing with a dynamic environment requires us to attend to where and when relevant information will appear. This ability is needed in our everyday activities, like speaking, playing, and appreciating music, and it is only possible because we can process and use temporal information across a wide range of intervals (Buhusi and Meck, 2005). The perception of time can be relative, beat-based timing, or absolute, duration-based timing (McAuley and Jones, 2003; Grahn and McAuley, 2009; Teki et al., 2012). It is traditionally argued that beat-based time perception allows prediction because intervals are encoded in relation to a regular beat interval (Winkler et al., 2009), while duration-based timing does not since it refers to the measurement of absolute interval lengths (Teki et al., 2012). More recent perspectives question whether prediction is absent in duration-based systems. Bouwer et al. (2020) found that beat- and duration-based timing mechanisms contribute to form expectations in ways that overlap at least partly. Additionally, Breska and Ivry (2018) argued that temporal prediction can be generated from irregular sequences if the duration of the intervals is known, for example, when there is previous exposure to those durations. Whatever the engagement of prediction in the two systems, the experimental tasks used to measure duration-based timing are clearly different from those used for beat-based timing (e.g., detect regularity of beat, synchronize with a beat). In the perceptual domain, one basic method to measure duration perception is to present participants a time interval and ask them to estimate how long it lasts—a method called time estimation. As for time production, one common method is to present time intervals and ask participants to reproduce these by clapping, tapping, or other motor action (Grondin et al., 2018).

Traditionally, beat-based and duration-based timing systems have been regarded as dissociated systems. Teki et al. (2011) provided evidence that the olivocerebellar system mediates absolute, duration-based timing while the striato-thalamo-cortical system subserves relative, beat-based timing. Some studies found that the basal ganglia and the supplementary motor area (SMA) respond more to beat-based than duration-based systems (Geiser et al., 2012; Grahn and Rowe, 2013; Li et al., 2019). However, in a more recent study, Teki et al. (2012) proposed a unified model of perceptual timing based on the common activation of the striato-thalamo-cortical and olivocerebellar networks and their strong connection with each other, suggesting that time is represented in a distributed manner. The connectivity between the two networks occurs through multiple loops, enabling them to operate simultaneously to achieve optimal precision. Critically, an asymmetrical relationship seems to exist between them, with the beat-based network serving as the primary timing mechanism, which is further refined by the duration-based network. This suggests that the olivo-cerebellar network may function as an error-correction device, continuously adjusting the internal temporal representation based on external signals. The impact of this network would be less significant when dealing with regular and predictable sequences of time intervals (De Pretto and James, 2015). Furthermore, a large body of neuroimaging studies indicated the involvement of the premotor cortex, the SMA, the cerebellum, and the basal ganglia in both beat- and duration-based perception (Koch et al., 2003; Jones et al., 2004; Kim and Zauberman, 2009; Chiba et al., 2015; Gouvêa et al., 2015). In sum, there seems to be some form of association between beat- and duration-based systems, wherein the former provides input to the latter.

Engagement with a regular beat boosts various dimensions of cognition and action (Grube and Griffiths, 2009; Grube et al., 2010). Ready et al. (2019) found that good beat perceivers were better in their gait when instructed to synchronize with cues compared to when no synchronization was required. The presence of a beat also enhances the perception of time-unrelated features of stimuli. When a sound sequence contains temporal regularity (an underlying beat), the perception of tones and sensory processing are improved (Jones et al., 2002; Ellis and Jones, 2010; Sanabria et al., 2011). This phenomenon can be explained by the temporal expectation (knowing when a new event will come) that can be built from the structure of a periodic sound (London, 2004). Geiser et al. (2012) investigated the neural substrates underlying perceptual facilitation by regular temporal context. Participants performed an intensity discrimination task while listening to temporally regular or irregular sequences. The authors found better performance in discrimination tasks when subjects listened to regular than irregular tone sequences.

Is it known that several factors can influence duration perception, like the duration of an interval (Rammsayer, 2014; Rammsayer et al., 2015), stimulus modality and many other psychophysical aspects of the interval like pitch, frequency, filled vs. empty interval, type of task (Penney and Tourret, 2005; van Wassenhove, 2009; Merchant et al., 2015). Given that beat- and duration-based timing share neural resources, and that the presence of beat information enhances cognition and action at various levels, could it be that motor reproduction (i.e., the ability to estimate and reproduce time intervals using motor skills like finger tapping or playing a drum) is influenced by internalized beat cues? To answer this question, Daikoku et al. (2018) examined how time intervals are reproduced in three conditions: with no internalized cue, with an internalized cue with a beat, and with an internalized cue without a beat. Results showed that the reproduction of intervals above 5.6 s is more accurate when internalized beat cues are used as an aid than when these are not, with decreased accuracy for absent beat cues appearing as underestimation (time intervals judged as shorter than they really were). According to Treisman (1963), there is a tendency to overestimate short intervals and underestimate long ones. To sum up, when invited to use beat cues for interval reproduction, subjects benefit from it above a certain interval length. Thus, the abilities to perceive a beat, synchronize with it, and reproduce the time information through motor actions or movements have various implications in daily activities. Although reproduction engages perception, to the best of our knowledge, few studies investigated the use of beat cues as a potential aid in a pure duration perception task.

The present study was designed to investigate whether duration perception is enhanced by preceding beat cues and, thus, investigate further the possible cooperation between duration-based and beat-based timing systems from the viewpoint of beat as the primary system that feeds duration-based processing. To that end, we asked participants to estimate the duration of auditory time intervals with vs. without preceding beat cues. In the cue condition, participants listened to a sequence of beats, the sequence stopped, and then the target interval was presented. In the no-cue condition, participants were exposed to a silent period equivalent in length to that of the beat sequence in the cue condition.

Our design implied that preceding beat cues would be internalized by participants and they would use them as an aid to estimate the intervals’ duration after they were removed. Such an assumption is backed by various research findings, including those centered on the idea of entrainment (or synchronization of brain oscillations with a beat, see Nozaradan et al., 2011) that persists after the beat ceases. Fujioka et al. (2010, 2012) showed that neural oscillations in motor-related areas elicited by auditory beats can still be maintained through the subjective imagination of the beat. In the same vein, Cheng et al. (2022), investigated how humans process hierarchical temporal information in music rhythm using the paradigm of meter imagination and the involvement of motor system in this process. They observed that neural responses to imagined meter were present in both auditory and motor sources, even in the absence of acoustic cues. The study extends Nozaradan et al. (2011) findings that a mental representation of a metric structure can induce neural entrainment at the same frequency as the given metric. Despite convergent evidence that entrainment may be sustained after the external input disappears (internalized beat), contrary reports of entrainment decay are also available (Cheng et al., 2022).

In our study, better time estimation performance with (than without) preceding beat cues would indicate that these cues were responsible for improving duration perception, and, thus, that they were internalized in the first place. However, if we had no cue effect, we would not be able to decide whether participants simply did not internalize the cue or, alternatively, if they internalized the cue but did not use it to aid the estimation. To test this, we manipulated the interference within the interval to be judged (a short white noise sequence). The manipulation consisted of presenting each interval under three conditions: without any interference, with beat-related, and with beat-unrelated interference. In the scenario of cues enhancing time estimation, facilitating effects of beat-related interference and/or disruptive effects of beat-unrelated interference (vs. no interference) with cues would strengthen the idea that cues were used as an aid. In the alternative scenario of null or negative effects of cue on time estimation, potential effects of interference—whatever the direction—would indicate that the beat had been internalized but not properly used. For comparison with Daikoku et al. (2018), we analyzed both estimation accuracy and the type of error (underestimation vs. overestimation) across interval duration. We hypothesized that beat cues would aid time estimation for longer intervals, namely by reducing the typical underestimation of these.

## Materials and methods

### Participants

One hundred and fifty-five adults took part in this study (*M*_*age*_ = 26, SD_*age*_ = 10.9; *M*_*schooling*_ = 16, *SD*_*schooling*_ = 1.6; *M*_*musictraining*_ = 4, *SD*_*musictraining*_ = 3.7). All participants were naïve to the purpose of the study, had normal or corrected-to-normal vision, and did not report neurological, motor, or hearing disorders. The study was approved by the local ethics committee (2021/06-07b) and all participants signed informed consent according to the Declaration of Helsinki.

### Stimulus materials

**FIGURE 1:**
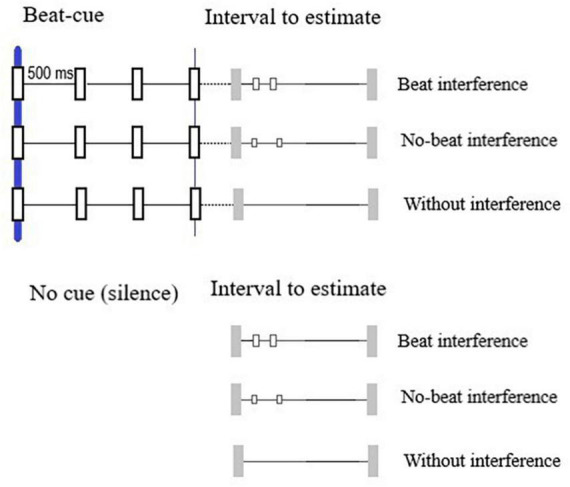
Intervals to estimate preceding by beat cues and no-cues. For each condition, intervals were presented under three versions: without interference, with beat interference, and non-beat interference.

We created 20 auditory intervals ranging between 500 and 10,000 ms with increments of 500 ms (Figure 1). Intervals were defined by two 1,350 Hz beeps with length of 70 ms each. Each interval was presented under three different versions: without interference, i.e., only the onset and offset beeps; with beat interference, i.e., with white noise events lasting 40 ms presented 250 ms and 500 ms after the interval onset; and with non-beat interference—with the same white noise events but presented at non-beat-compatible rates (100 and 500 ms after the onset). These three different versions of the interval set (20 + 20 + 20 = 60) were presented once–under the cue condition (preceded by 2 s of beat cue sequence) and under the no-cue condition (preceded by 2 s of silence). Therefore, the set of 20 intervals was presented six times—3 interference-related conditions (with beat-interference, without beat-interference, and without interference) × 2 cue-related conditions (cue and no-cue), totalizing 120 trials per participant.

### Procedure

Participants were asked to estimate the duration of each interval by pressing the corresponding number keys on the computer keyboard (i.e., for 1 s response, the related key was 1) and also to choose the lowest interval when they thought it was between two values (e.g., respond 2 s when the interval was between 2 and 3 s). The reason to provide this instruction and not the alternative [respond 3 s when the interval was between 2 and 3 s was that the former is more intuitive and mirrors common language (e.g., people say to 2 and a half seconds instead of 3 minus half seconds)]. For 500 ms and 10 s responses, participants would press, respectively, the “0” and “p” keys. After completing the consent form, we explained to participants that, in some situations, they would listen to two beeps, and in others, they would also listen to two noises between the beeps. At the end of each example, participants were asked to provide their response, and the exact time estimation was shown right after it. The examples were given for both cue- and no-cue conditions. During the task, participants listened to one block of stimuli for each condition: with and without preceding beat cue sequences. Half the participants went through the cue condition first, and the other half started with the no-cue condition. Within each block, stimuli were randomized for type of interference and interval duration in seconds. In the cue-condition, participants were instructed to listen to a rhythmic sequence before estimating the duration of time intervals. They were told that the sequence was composed of four beeps with 500 ms inter-onset interval, lasting 2 s in total. In the no-cue condition, they waited 2 s in silence, and then the time interval was presented.

### Data analysis

The data were analyzed using a linear mixed effects model based on the lme4 package (Bates et al., 2015) in R (R Core Team, 2012). In the first analysis, we examined response error as a measure of accuracy. The error was calculated as the absolute difference (positive values only) between the target duration and the response. For non-integer intervals (e.g., 1.5, 2.5), the target value was the first digit (here, 1 and 2). The predictors of interest, interval duration (11 levels, from < 1 to 10 s), beat cue (yes vs. no), and type of interference (three levels: beat, non-beat and none) were entered as fixed factors while participants were treated as random intercepts. We tested the main effects of the three predictors—interval duration, beat cue, and type of interference and all possible interactions among them. Complementary to this main analysis, we repeated the model, adding block order as covariate (cue first vs. no-cue first) to check whether this might have affected the results. For exploratory purposes, we also checked whether participants’ responses were more variable (standard deviation and coefficient of variance of responses) in the no-cue than in the cue condition.

To address the type of error—under vs. overestimation—and complete the comparison with production-related results (Daikoku et al., 2018), we ran a second analysis wherein we viewed error in a directional perspective: as either positive (participant indicates duration longer than the target) or negative (duration shorter than the target). Here, we used one-sample two-tailed *t*-tests against zero or their non-parametric equivalent to determine whether responses deviated positively (overestimation) or negatively (underestimation) from the target. We started at < 1 s intervals, and proceeded until we had the first significant underestimation. Since non-integer intervals (1.5 s, 2.5 s., etc.) were more exposed to inconsistent responses across participants (despite the instructions, some could choose the upper limit and others the lower one), we ran a control analysis to check whether these results survived when only integers (the most reliable intervals) were considered.

According to sensitivity power analyses carried out with G*Power (Faul et al., 2007), the minimum effects sizes (three factors) that our tests were able to detect reliably with 80% power (alpha = 0.05) were in the small range (*f* < 0.10; *f* = 0.05 in our case).

## Results

### Estimation error

**FIGURE 2:**
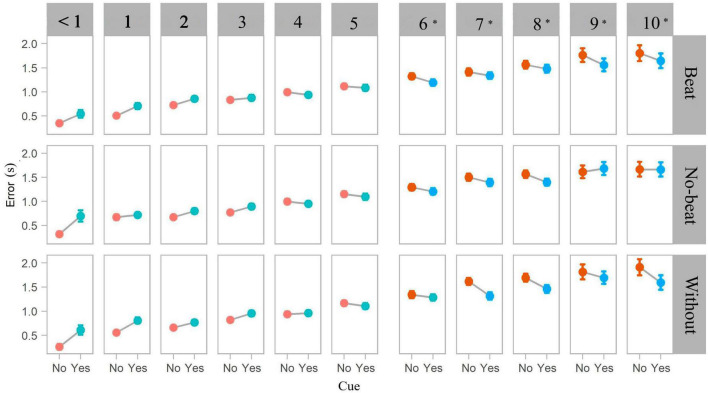
Error estimation [absolute value of (target interval—response)] as a function of interval duration (< 1–10 s), cue condition (cue vs. no-cue), and type of interference (beat, no-beat, and none). *p < 0.05.

The mixed-effects regression showed a significant main effect of interval duration and a marginal effect of cue (*p* = 0.095, see Supplementary Table A), while type of interference yielded non-significant effects (*p* = 0.443). The significant interaction between interval duration and cue showed that the presence of a beat cue improves time estimation in intervals ranging from 6 to 10 s (all *p*s < 0.005, Figure 2), while there was no significant effect of beat cues in intervals ranging from < 1 to 5 s (Supplementary Table A). The interactions between cue (yes vs. no) and type of interference, between interval duration and type of interference, as well as those engaging the three predictors (cue × interference × duration) yielded no significant effects either (all *p*s > 0.05). The order in which intervals were presented (beat-cue condition prior to the no-cue condition) yielded non-significant effects on estimation error (all *p*s > 0.05, Supplementary Table B). Additionally, the variability of estimation responses was similar across cue conditions for all durations (Supplementary Table C), suggesting that beat cues do not increase or decrease the consistency of responses across participants.

### Under vs. overestimation

**FIGURE 3:**
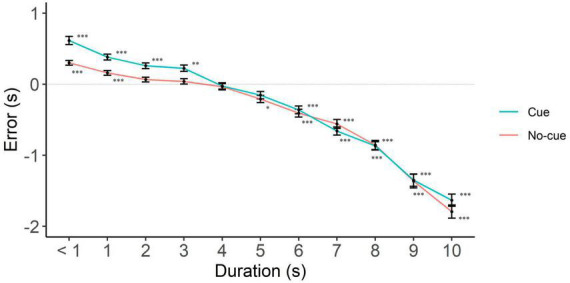
Directional error (target interval—response) as a function of cue and interval duration. One-sample two-tailed *t*-test analyses started at < 1 s intervals and proceeded until we had the first significant deviation from zero. Positive, negative and zero values indicate overestimation, underestimation, and no error, respectively. *p < 0.05; **p < 0.01; ***p < 0.001.

To examine the direction of the error, we collapsed interference levels and analyzed the data separately per cue level only, since cue was the only predictor that interacted with interval duration. We put focus on the interval range where cue had an effect, i.e., 6 to 10 s. In line with the literature on interval production, we found underestimation only (participants responding with a shorter interval than the one presented, Figure 3 and Table 1). In the cue condition, underestimation started at 6 s and remained till the longest interval. Consistent with the benefit of cues to performance, underestimation started earlier in the no-cue condition, from 5 s trials onward.

**TABLE 1:** One sample *t*-test against zero for error estimation per level of cue (cue vs. no-cue) and interval duration (< 1 to 10 sec).

		No-cue				Cue	
**Interval duration (s)**	**Test**	**Statistic**	***P*-value**	**Interval duration**	**Test**	**Statistic**	***P*-value**
< 1	Wilcoxon	2,518	**< 0.001**	< 1	Wilcoxon	3,403	**< 0.001**
1	Student	52,795	**< 0.001**	1	Wilcoxon	7,555	**< 0.001**
2	Student	1.1336	0.258	2	Wilcoxon	6,095	**< 0.001**
3	Student	0.4991	0.618	3	Wilcoxon	6,143	**0.04**
4	Student	−0.4435	0.658	4	Wilcoxon	4,711	0.64
5	Student	−2.17	**0.031**	5	Wilcoxon	4,452	0.09
6	Student	−3.6287	**< 0.001**	6	Wilcoxon	3,395	**< 0.001**
7	Student	−4.2488	**< 0.001**	7	Wilcoxon	2,627	**< 0.001**
8	Wilcoxon	2,409	**< 0.001**	8	Wilcoxon	1,656.5	**< 0.001**
9	Wilcoxon	1,009.5	< 0.001	9	Wilcoxon	780.5	**< 0.001**
10	Wilcoxon	0	**< 0.001**	10	Student	−12.662	**< 0.001**

Significant *p*-values are in bold.

**FIGURE 4:**
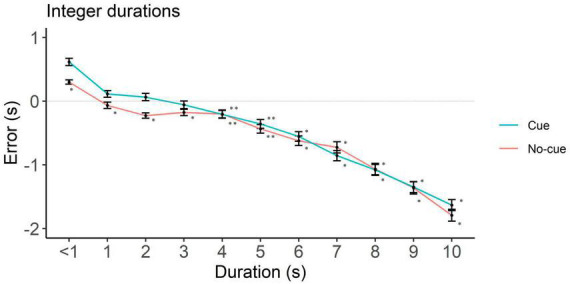
Directional error (target interval—response) as a function of cue and duration for integer durations only. One-sample two-tailed *t*-test analyses started at < 1 s durations and proceeded until we had the first significant underestimation. Positive, negative and zero values indicate overestimation, underestimation and no error, respectively. *p < 0.001, **p < 0.05.

**FIGURE 5:**
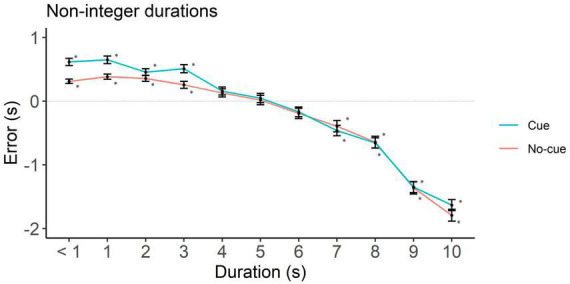
Directional error (target interval—response) as a function of cue and duration for non-integer durations only. One-sample two-tailed *t*-test analysis started at < 1 s durations and proceeded until we had the first significant underestimation. Positive, negative and zero values indicate overestimation, underestimation and no error, respectively. *p < 0.001.

To rule out potential influences from requesting participants a down rounded response in case they perceived an intermediate interval, we analyzed underestimation in integer and non-integer intervals separately. In integers, the cue condition showed underestimation starting at 4 s (*p* = 0.01) and the no-cue at 1 s (*p* < 0.001, Figure 4 and Table 2). In non-integers underestimation started at 7 s in both cue conditions (*p* < 0.001, Figure 5 and Table 2).

**TABLE 2:** One sample *t*-test against zero for error estimation per level of cue (cue vs. no-cue), interval duration (< 1 to 10 sec) and target interval (integer vs. non-integer).

		No-cue				Cue	
**Integer duration (s)**	**Test**	**Statistic**	***P*-value**	**Integer duration (s)**	**Test**	**Statistic**	***P*-value**
1	Wilcoxon	7,776	**< 0.001**	1	Student	1.5021	0.067
2	Wilcoxon	2,090.5	**< 0.001**	2	Student	0.79828	0.425
3	Wilcoxon	2,945	**< 0.001**	3	Student	−0.787	0.432
4	Wilcoxon	3,143	**< 0.001**	4	Wilcoxon	3,238	**0.012**
5	Student	−4.403	**< 0.001**	5	Wilcoxon	2,753	**0.003**
6	Student	−5.4242	**< 0.001**	6	Wilcoxon	2,071.5	**< 0.001**
7	Student	−5.2801	**< 0.001**	7	Wilcoxon	1,490	**< 0.001**
8	Wilcoxon	1,590.5	**< 0.001**	8	Student	−8.7823	**< 0.001**
9	Wilcoxon	1,009.5	**< 0.001**	9	Student	−10.289	**< 0.001**
10	Wilcoxon	0	**< 0.001**	10	Student	−12.662	**< 0.001**
		**No-cue**				**Cue**	
**Non-integer duration (s)**	**Test**	**Statistic**	***P*-value**	**Non-integer duration (s)**	**Test**	**Statistic**	***P*-value**
< 1	Wilcoxon	2,518	**< 0.001**	< 1	Wilcoxon	3,403	**< 0.001**
1	Wilcoxon	5,746.5	**< 0.001**	1	Wilcoxon	6,231	**< 0.001**
2	Wilcoxon	5,594	**< 0.001**	2	Wilcoxon	5,512	**< 0.001**
3	Wilcoxon	5,466.5	**< 0.001**	3	Wilcoxon	6,747	**< 0.001**
4	Wilcoxon	5,154.5	0.213	4	Wilcoxon	5,160	0.111
5	Student	0.98853	0.235	5	Wilcoxon	4,903.5	0.489
6	Wilcoxon	4,373.5	0.241	6	Wilcoxon	3,315.5	0.124
7	Student	−2.9083	**< 0.001**	7	Wilcoxon	2,692.5	**< 0.001**
8	Wilcoxon	2,854.5	**< 0.001**	8	Wilcoxon	2,025	**< 0.001**

Significant *p*-values are in bold.

For the short interval range, we found instances of overestimation. When considering all interval durations, the estimated interval (response) was significantly longer than the one presented up to 4 s in the cue condition and up to 1 s in the no-cue condition. Note that, despite the more extended presence of overestimation in the cue condition, the main analysis—which prevails when it comes to drawing conclusions—showed that the estimation error was equivalent to that of the no-cue condition in the < 1 to 5 s range. From this viewpoint, overestimation results are irrelevant to our goal.

In sum, longer intervals—those in which we found cue effects—were marked by underestimation. Underestimation was present in integer as well as non-integer long intervals, even though it was less extensive in the latter.

## Discussion

The current study examined whether beat cues aid duration-based perception. To that end, we examined the effects of cue (yes vs. no) and interference during interval presentation (no-beat vs. beat vs. no interference regarding beat cues) on estimation error (absolute deviation response-target) and error direction (overestimation vs. underestimation). Beat cues aided time estimation in longer intervals: from 6 s intervals onward (6 s included), time estimation was better in the cue than in the no-cue condition, and underestimation began later in the cue condition (6 s), compared to non-cue (5 s). These results partly replicate previous evidence that beat cues aid interval reproduction in intervals longer than 5.6 s, specifically by decreasing underestimation (Daikoku et al., 2018). The idea that beat cues aid duration perception is in line with the unified model proposed by Teki et al. (2012), where timing (i.e., perception and production), involves beat-based system activation followed by duration-based system activation.

Apart from cue effects, participants’ responses to short vs. long intervals were in line with the literature. The pattern of over- followed by underestimation is in line with a well-established finding in the timing domain: that constant error—measured by subtracting the mean time estimation from the standard duration—is positive in short intervals but negative in long ones (Woodrow, 1930, 1951; Woodworth and Schlosberg, 1954; Chun et al., 2018). As for the cutoff interval of 6 s we found, it overlaps partly with major cutoff references available in the timing literature. While Fraisse (1984) and later Pöppel (1997, 2004) described a post sensory-integration window with a cutoff point at 3 s, where underestimation would start to occur, Noulhiane et al.’s (2009) findings suggested that cuttoff points should be framed according to the duration range under consideration. For instance, in duration sets limited to 1–5.5 s, the cutoff would be located around 3 s, but it would shift to 5 s with durations between 1 and 10 s.

Contrary to our expectation, interference had no effects. It cannot be excluded that the lack of effects is a result of poor methodological choices, namely the position of the interfering events (possibly too early), their timbral characteristics (here we used white noise, which does not have a sharp attack and thus may not favor precise onset detection), or the low contrast between beat and non-beat interference: beat interference was presented 250 and 500 ms after the interval onset, and non-beat interference occurred 100 and 500 ms after the onset. Future research is necessary to investigate how these choices might modulate the role of no-beat vs. beat interference in the paradigm we used.

Another variable that deserves future research attention is the length of the cue sequence: could a longer beat sequence enhance the effect we saw? Daikoku et al. (2018) have shown that increased exposure to beats (13 beats in their study) can lead to stable internalization and induce accurate interval reproduction in long durations (over 5.6 s). Also, concerning the cue sequence, it would be important to investigate how different inter-onset-intervals in beat sequences (here, IOI of 500 ms) might modulate the impact of beat cues on time estimation. Drake and Botte (1993) found that the highest sensitivity for tempo discrimination ranges between 300 to 800 ms and increased IOI worsens time performance, possibly reflecting a boundary between automatic (IOI of 300 ms) and more controlled processing (IOI of 3,500 ms) proposed by Xu et al. (2021). For instance, would accuracy in time estimation increase with a suboptimal (100 ms) or supraoptimal (1,000 ms) beat cue?

Finally, we mentioned the possibility of entrainment of brain oscillations to beat cues as a neural substrate of internalized beat cues, but we did not collect Electroencephalogram (EEG) responses to substantiate such claim. It is unlikely that something other than internalizing beat cues condition (other processes would manifest also in the no-cue condition), and, thus, it is possible that oscillatory entrainment took place in participants’ brains. In this sense, the logical follow-up would be an EEG study using the same paradigm.

Despite these limitations, our findings contribute to expand current knowledge on the link between beat and duration mechanisms, thus challenging more traditional views of dissociation between the two. The idea of an overlapping system that uses the same networks but with varying intensity, depending on the regularity of the input, could be considered a principle of parsimony. In everyday life, where regular and irregular contexts constantly alternate, employing the same networks becomes more efficient and economical. To our knowledge, our study was the first to show that purely perceptual tasks like interval estimation benefit from beat cues, by specifically decreasing underestimation of longer intervals. Critically, our study points to similarities between beat effects on duration production (Daikoku et al., 2018) and beat effects on pure perception (the current study), strengthening the idea of beat subserving duration processing, and paving the way for a novel perspective on timing skills. Could it be, for instance, that problems related to duration perception are rooted in beat processing impairments? Future studies might bring responses to these questions.

## Data availability statement

The datasets presented in this study can be found in online repositories. The names of the repository/repositories and accession number(s) can be found below: https://osf.io/27k8v/.

## Ethics statement

The studies involving humans were approved by the Ethics Committee of Faculty of Psychology and Educational Sciences of University of Porto 2021/06-07b. The studies were conducted in accordance with the local legislation and institutional requirements. The participants provided their written informed consent to participate in this study.

## Author contributions

NT recruited subjects, collected the data, and wrote the manuscript. SS devised the paradigm. SS and NT analyzed the data and discussed the results. All authors contributed to the article, refined the manuscript and approved the submitted version.
