# Supplementary material for: Beat cues facilitate time estimation at longer intervals
Source: Front Psychol. 2023 Sep 29;14:1130788. doi: 10.3389/fpsyg.2023.1130788 (PMC10576433; doi:10.3389/fpsyg.2023.1130788)
Supplement: Supplementary file 1 [file Data_Sheet_1.docx]

Supplementary Material

Beat Cues Facilitate Time Estimation for Longer Intervals

Nathércia L. Torres*, São Luís Castro, Susana Silva

*** Correspondence:** Corresponding Author: up201602198@edu.fpce.up.pt

**Supplementary Table A.** *Mixed-effects regression of cue, type of interference, and interval duration effects on estimation error.*

|  |  | |  | **ERROR** |  |
| --- | --- | --- | --- | --- | --- |
| *Predictors* |  | | *Estimates* | *CI* | *P* |
| Intercept | |  | 0.34 | 0.16 – 0.53 | < .001 |
| Cue [yes] | |  | 0.20 | -0.03 – 0.42 | 0.095 |
| Interference [no] | |  | -0.09 | -0.32 – 0.14 | 0.443 |
| Interference [non-beat] | |  | -0.03 | -0.10 – 0.06 | 0.606 |
| Duration [<1s] | |  | 0.19 | 008 – 0.30 | **0.001** |
| Duration [2s] | |  | 0.38 | 0.18 – 0.58 | **<0.001** |
| Duration [3s] | |  | 0.49 | 0.29 – 0.69 | **<0.001** |
| Duration [4s] | |  | 0.65 | 0.45 – 0.85 | **<0.001** |
| Duration [5s] | |  | 0.77 | 0.57 – 0.97 | **<0.001** |
| Duration [6s] | |  | 0.98 | 0.78 – 1.18 | **<0.001** |
| Duration [7s] | |  | 1.07 | 0.87 – 1.27 | **<0.001** |
| Duration [8s] | |  | 1.22 | 1.02 – 1.42 | **<0.001** |
| Duration [9s] | |  | 1.42 | 1.19 – 1.65 | **<0.001** |
| Duration [10s] | |  | 1.46 | 1.23 – 1.68 | **<0.001** |
| Cue [yes]*duration [< 1s] | |  | 0.01 | -0.27 – 0.29 | 0.952 |
| Cue [yes]*duration [2s] | |  | -0.07 | -0.35 – 0.21 | 0.642 |
| Cue [yes]*duration [3 s] | |  | -0.15 | -0.43 – 0.13 | 0.295 |
| Cue [yes]*duration [4 s] | |  | -0.25 | -0.53 – 0.02 | 0.074 |
| Cue [yes]*duration [5 s] | |  | -0.22 | -0.51 – 0.06 | 0.115 |
| Cue [yes]*duration [6 s] | |  | -0.33 | -0.61 – -0.05 | **0.022** |
| Cue [yes]*duration [7 s] | |  | -0.27 | -0.55 – 0.01 | **0.058** |
| Cue [yes]*duration [8 s] | |  | -0.28 | -0.56 – 0.00 | **0.051** |
| Cue [yes]*duration [9 s] | |  | -0.40 | -0.72 – -0.07 | **0.016** |
| Cue [yes]*duration [10 s] | |  | -0.35 | -0.67 – -0.03 | **0.034** |
| Cue [yes]* interference [no-beat] | |  | 0.18 | -0.14 – 0.50 | 0.275 |
| Cue [yes]*interference [none] | |  | 0.17 | -0.15 – 0.49 | 0.306 |
| Interference [no-beat] *duration [<1s] | |  | 0.19 | -0.09 – 0.47 | 0.179 |
| Interference [none] *duration [<1s] | |  | 0.14 | -0.14 – 0.42 | 0.325 |
| Interference [no-beat]*duration [2s] | |  | -0.03 | -0.31 – 0.25 | 0.840 |
| Interference [none]*duration [2s] | |  | 0.02 | -0.26 – 0.30 | 0.877 |
| Interference [no-beat]*duration [3s] | |  | -0.04 | -0.32 – 0.25 | 0.805 |
| Interference [none]*duration [3s] | |  | 0.08 | -0.20 – 0.36 | 0.592 |
| Interference [no-beat]*duration [4s] | |  | 0.03 | -0.25 – 0.31 | 0.858 |
| Interference [none]*duration [4s] | |  | 0.03 | -0.25 – 0.31 | 0.824 |
| Interference [no-beat]*duration [5s] | |  | 0.06 | -0.22 – 0.34 | 0.654 |
| Interference [none]*duration [5s] | |  | 0.14 | -0.14 – 0.42 | 0.315 |
| Interference [no-beat]*duration [6s] | |  | -0.01 | -0.29 – 0.27 | 0.964 |
| Interference [none]*duration [6s] | |  | 0.11 | -0.17 – 0.39 | 0.447 |
| Interference [no-beat]*duration [7s] | |  | 0.07 | -0.04 – 0.17 | 0.195 |
| Interference [none]*duration [7s] | |  | 0.09 | -0.02 – 0.19 | 0.097 |
| Interference [no-beat]*duration [8s] | |  | 0.03 | -0.25 – 0.31 | 0.858 |
| Interference [none]*duration [8s] | |  | 0.22 | -0.06 – 0.50 | 0.128 |
| Interference [no-beat]*duration [9s] | |  | -0.12 | -0.45 – 0.20 | 0.461 |
| Interference [none]*duration [9s] | |  | 0.14 | -0.18 – 0.46 | 0.394 |
| Interference [no-beat]*duration [10s] | |  | -0.11 | -0.43 – 0.21 | 0.510 |
| Interference [none]*duration [10s] | |  | 0.20 | -0.13 – 0.52 | 0.229 |
| Cue [yes]*interference [none]*duration [1s] | |  | -0.12 | -0.52 – 0.28 | 0.551 |
| Cue [yes]*interference [no-beat]*duration [1s] | |  | -0.34 | -0.74 – 0.06 | 0.093 |
| Cue [yes]*interference [none]*duration [2s] | |  | -0.19 | -0.58 – 0.21 | 0.354 |
| Cue [yes]*interference [no-beat]*duration [2s] | |  | -0.19 | -0.58 – 0.21 | 0.353 |
| Cue [yes]*interference [none]*duration [3s] | |  | -0.08 | -0.48 – 0.32 | 0.691 |
| Cue [yes]*interference [no-beat]*duration [3s] | |  | -0.11 | -0.51 – 0.29 | 0.589 |
| Cue [yes]*interference [none]*duration [4s] | |  | -0.09 | -0.48 – 0.31 | 0.668 |
| Cue [yes]*interference [no-beat]*duration [4s] | |  | -0.17 | -0.57 – 0.22 | 0.393 |
| Cue [yes]*interference [none]*duration [5s] | |  | -0.20 | -0.60 – 0.19 | 0.311 |
| Cue [yes]*interference [no-beat]*duration [5s] | |  | -0.22 | -0.61 – 0.18 | 0.288 |
| Cue [yes]*interference [none]*duration [6s] | |  | -0.10 | -0.49 – 0.30 | 0.638 |
| Cue [yes]*interference [no-beat]*duration [6s] | |  | -0.14 | -0.54 – 0.26 | 0.489 |
| Cue [yes]*interference [none]*duration [7s] | |  | -0.39 | -0.79 – 0.00 | 0.053 |
| Cue [yes]*interference [no-beat]*duration [7s] | |  | -0.22 | -0.61 – 0.18 | 0.285 |
| Cue [yes]*interference [none]*duration [8s] | |  | -0.32 | -0.71 – 0.08 | 0.118 |
| Cue [yes]*interference [no-beat]*duration [8s] | |  | -0.27 | -0.66 – 0.13 | 0.187 |
| Cue [yes]*interference [none]*duration [9s] | |  | -0.09 | -0.55 – 0.37 | 0.705 |
| Cue [yes]*interference [no-beat]*duration [9s] | |  | 0.08 | -0.37 – 0.54 | 0.717 |
| Cue [yes]*interference [none]*duration [10s] | |  | -0.33 | -0.79 – 0.13 | 0.156 |
| Cue [yes]*interference [no-beat]*duration [10s] | |  | -0.03 | -0.49 – 0.42 | 0.885 |
| **Random effects** | |  |  |  |  |
| σ^2^ | |  | 1.06 |  |  |
| τ_00_ _subject_ | |  | 0.35 |  |  |
| ICC | |  | 0.25 |  |  |
| N _subject_ | |  | 155 |  |  |
| Observations | |  | 17763 |  |  |
| Marginal R^2^/Conditional R^2^ | |  | 0.091/0.314 |  |  |

**Supplementary Table B**. *Mixed-effects regression of cue, type of interference, interval duration and block order (cue first vs.. no-cue first) effects on estimation error.*

|  |  |  | **ERROR** |  |
| --- | --- | --- | --- | --- |
| *Predictors* |  | *Estimates* | *CI* | *P* |
| Cue [Yes] |  | 0.14 | -0.19 – 0.58 | **0.409** |
| Intercept |  | 0.31 | 0.05 – 0.58 | **0.021** |
| Duration [1 s] |  | 0.24 | -0.04 – 0.52 | 0.094 |
| Duration [2 s] |  | 0.44 | 0.15 – 0.72 | **0.002** |
| Duration [3 s] |  | 0.54 | 0.26 – 0.82 | **<0.001** |
| Duration [4 s] |  | 0.75 | 0.47 – 1.03 | **<0.001** |
| Duration [5 s] |  | 0.88 | 0.60 – 1.16 | **<0.001** |
| Duration [6 s] |  | 1.04 | 0.76 – 1.32 | **<0.001** |
| Duration [7 s] |  | 1.15 | 0.87 – 1.43 | **<0.001** |
| Duration [8 s] |  | 1.23 | 0.95 – 1.52 | **<0.001** |
| Duration [9 s] |  | 1.71 | 1.39 – 2.04 | **<0.001** |
| Duration [10 s] |  | 1.66 | 1.34 – 1.99 | **<0.001** |
| Block order [cue–no cue] |  | 0.06 | -0.31 – 0.44 | 0.734 |
| Interference [no-beat] |  | 0.06 | -0.26 – 0.39 | 0.695 |
| Interference [none] |  | -0.15 | -0.48 – 0.17 | 0.352 |
| Cue [yes]*duration [1 s] |  | -0.11 | -0.51 – 0.28 | 0.580 |
| Cue [yes]*duration [2 s] |  | -0.10 | -0.50 – 0.29 | 0.612 |
| Cue [yes]*duration [3 s] |  | -0.15 | -0.55 – 0.24 | 0.446 |
| Cue [yes]*duration [4 s] |  | -0.25 | -0.65 – 0.15 | 0.214 |
| Cue [yes]*duration [5 s] |  | -0.20 | -0.60 – 0.19 | 0.314 |
| Cue [yes]*duration [6 s] |  | -0.32 | -0.72 – 0.08 | 0.113 |
| Cue [yes]*duration [7 s] |  | -0.24 | -0.64 – 0.16 | 0.240 |
| Cue [yes]*duration [8 s] |  | -0.25 | -0.64 – 0.15 | 0.223 |
| Cue [yes]*duration [9 s] |  | -0.70 | -1.16 – -0.24 | **0.003** |
| Cue [yes]*duration [10 s] |  | -0.55 | -1.01 – -0.09 | **0.018** |
| Cue [yes]*block order [Cue–No cue] |  | 0.12 | -0.34 – 0.57 | 0.616 |
| Duration [1 s]*block order [cue–no cue] |  | -0.16 | -0.56 – 0.23 | 0.416 |
| Duration [2 s]*block order [cue–no cue] |  | -0.11 | -0.50 – 0.29 | 0.600 |
| Duration [3 s]*block order [cue–no cue] |  | -0.10 | -0.50 – 0.29 | 0.612 |
| Duration [4 s]*block order [cue–no cue] |  | -0.19 | -0.59 – 0.21 | 0.347 |
| Duration [5 s]*block order [cue–no cue] |  | -0.21 | -0.61 – 0.18 | 0.293 |
| Duration [6 s]*block order [cue–no cue] |  | -0.12 | -0.52 – 0.27 | 0.548 |
| Duration [7 s]*block order [cue–no cue] |  | -0.16 | -0.56 – 0.23 | 0.422 |
| Duration [8 s]*block order [cue–no cue] |  | -0.03 | -0.43 – 0.36 | 0.877 |
| Duration [9 s]*block order [cue–no cue] |  | -0.59 | -1.04 – -0.13 | **0.012** |
| Duration [10 s]*block order [cue–no cue] |  | -0.41 | -0.87 – 0.05 | 0.079 |
| Cue [Yes]*interference [no-beat] |  | 0.05 | -0.41 – 0.51 | 0.829 |
| Cue [Yes]*interference [none] |  | 0.25 | -0.21 – 0.71 | 0.287 |
| Duration [1 s]*  interference [no-beat] |  | 0.06 | -0.33 – 0.46 | 0.749 |
| Duration [2 s]*  interference [no-beat] |  | -0.16 | -0.56 – 0.24 | 0.424 |
| Duration [3 s]*  interference [no-beat] |  | -0.13 | -0.53 – 0.27 | 0.523 |
| Duration [4 s]*  interference [no-beat] |  | -0.10 | -0.50 – 0.29 | 0.609 |
| Duration [5 s]*  interference [no-beat] |  | -0.05 | -0.45 – 0.35 | 0.798 |
| Duration [6 s]*  interference [no-beat] |  | -0.06 | -0.46 – 0.34 | 0.774 |
| Duration [7 s]*  interference [no-beat] |  | 0.16 | -0.24 – 0.56 | 0.424 |
| Duration [8 s]*  interference [no-beat] |  | 0.01 | -0.39 – 0.40 | 0.974 |
| Duration [9 s]*  interference [no-beat] |  | -0.48 | -0.94 – -0.02 | **0.040** |
| Duration [10 s]*  interference [no-beat] |  | -0.29 | -0.75 – 0.17 | 0.223 |
| Duration [1 s]*  interference [none] |  | 0.17 | -0.23 – 0.56 | 0.410 |
| Duration [2 s]*  interference [none] |  | 0.02 | -0.37 – 0.42 | 0.906 |
| Duration [3 s]*  interference [none] |  | 0.08 | -0.31 – 0.48 | 0.684 |
| Duration [4 s]*  interference [none] |  | 0.06 | -0.34 – 0.45 | 0.781 |
| Duration [5 s]*  interference [none] |  | 0.19 | -0.21 – 0.59 | 0.343 |
| Duration [6 s]*  interference [none] |  | 0.25 | -0.15 – 0.65 | 0.215 |
| Duration [7 s]*  interference [none] |  | 0.36 | -0.03 – 0.76 | 0.073 |
| Duration [8 s]*  interference [none] |  | 0.31 | -0.09 – 0.71 | 0.126 |
| Duration [9 s]*  interference [none] |  | -0.05 | -0.51 – 0.41 | 0.818 |
| Duration [10 s]*  interference [none] |  | 0.36 | -0.10 – 0.82 | 0.122 |
| Block order [cue–no cue]*interference [no-beat] |  | -0.18 | -0.64 – 0.28 | 0.443 |
| Block order [cue–no cue]*interference [none] |  | 0.13 | -0.33 – 0.58 | 0.581 |
| Cue [yes]*duration [1 s] *block order [cue–no cue] |  | 0.24 | -0.32 – 0.80 | 0.402 |
| Cue [yes]*duration [2 s]  *block order [cue–no cue] |  | 0.07 | -0.49 – 0.63 | 0.803 |
| Cue [yes]*duration [3 s]  *block order [cue–no cue] |  | 0.01 | -0.55 – 0.57 | 0.975 |
| Cue [yes]*duration [4 s]  * block order [cue–no cue] |  | -0.01 | -0.57 – 0.55 | 0.978 |
| Cue [yes]*duration [5 s]  *block order [cue–no cue] |  | -0.04 | -0.60 – 0.52 | 0.880 |
| Cue [yes]*duration [6 s]  *block order [cue–no cue] |  | -0.01 | -0.57 – 0.55 | 0.966 |
| Cue [yes]*duration [7 s]  *block order [cue–no cue] |  | -0.07 | -0.62 – 0.49 | 0.817 |
| Cue [yes]*duration [8 s]  *block order [cue–no cue] |  | -0.06 | -0.62 – 0.49 | 0.824 |
| Cue [yes]*duration [9 s]  *block order [cue–no cue] |  | 0.60 | -0.04 – 1.25 | 0.068 |
| Cue [yes]*duration [10 s]  *block order [cue–no cue] |  | 0.40 | -0.24 – 1.05 | 0.223 |
| Cue [yes]*duration [1 s] *interference [no-beat] |  | -0.17 | -0.73 – 0.40 | 0.566 |
| Cue [yes]*duration [2 s] *interference [no-beat] |  | -0.03 | -0.59 – 0.53 | 0.917 |
| Cue [yes]*duration [3 s] *interference [no-beat] |  | -0.02 | -0.58 – 0.54 | 0.944 |
| Cue [yes]*duration [4 s] *interference [no-beat] |  | 0.04 | -0.53 – 0.60 | 0.898 |
| Cue [yes]*duration [5 s] *interference [no-beat] |  | -0.07 | -0.64 – 0.49 | 0.794 |
| Cue [yes]*duration [6 s] *interference [no-beat] |  | -0.03 | -0.59 – 0.54 | 0.930 |
| Cue [yes]*duration [7 s] *interference [no-beat] |  | -0.18 | -0.74 – 0.38 | 0.529 |
| Cue [yes]*duration [8 s] *interference [no-beat] |  | -0.14 | -0.71 – 0.42 | 0.620 |
| Cue [yes] * duration [9 s] * interference [no-beat] |  | 0.63 | -0.02 – 1.28 | 0.058 |
| Cue [yes]*duration [10 s] *interference [no-beat] |  | 0.48 | -0.17 – 1.13 | 0.145 |
| Cue [yes]*duration [1 s] *interference [no] |  | -0.14 | -0.70 – 0.42 | 0.625 |
| Cue [yes]*duration [2 s] *interference [no] |  | -0.25 | -0.81 – 0.31 | 0.389 |
| Cue [yes]*duration [3 s] *interference [no] |  | -0.16 | -0.72 – 0.40 | 0.572 |
| Cue [yes]*duration [4 s] *interference [no] |  | -0.13 | -0.69 – 0.43 | 0.657 |
| Cue [yes]*duration [5 s] *interference [no] |  | -0.27 | -0.83 – 0.29 | 0.346 |
| Cue [yes]*duration [6 s] *interference [no] |  | -0.19 | -0.75 – 0.37 | 0.513 |
| Cue [yes] *duration [7 s] *interference [no] |  | -0.43 | -0.99 – 0.13 | 0.136 |
| Cue [yes *duration [8 s] *interference [no] |  | -0.29 | -0.86 – 0.27 | 0.304 |
| Cue [yes]*duration [9 s] * interference [no] |  | 0.17 | -0.48 – 0.82 | 0.600 |
| Cue [yes]*duration [10 s] *interference [no] |  | -0.32 | -0.97 – 0.33 | 0.332 |
| Cue [yes]*block order [cue–no cue]*interference [no-beat] |  | 0.25 | -0.39 – 0.90 | 0.442 |
| Cue [yes]*block order [cue–no cue]*interference [none] |  | -0.16 | -0.81 – 0.48 | 0.626 |
| Duration [1 s]*block order [cue–no cue]*interference [no-beat] |  | 0.25 | -0.31 – 0.81 | 0.378 |
| Duration [2 s *block order [cue–no cue]*interference [no-beat] |  | 0.26 | -0.30 – 0.82 | 0.356 |
| Duration [3 s]*block order [cue–no cue]*interference [no-beat] |  | 0.19 | -0.37 – 0.75 | 0.513 |
| Duration [4 s]*block order [cue–no cue]*interference [no-beat] |  | 0.26 | -0.30 – 0.82 | 0.370 |
| Duration [5 s]*block order [cue–no cue]*interference [no-beat] |  | 0.23 | -0.33 – 0.79 | 0.422 |
| Duration [6 s]*block order [cue–no cue]*interference [no-beat] |  | 0.10 | -0.46 – 0.66 | 0.719 |
| Duration [7 s]*block order [cue–no cue]*interference [no-beat] |  | -0.10 | -0.66 – 0.46 | 0.729 |
| Duration [8 s]*block order [cue–no cue]*interference [no-beat] |  | 0.04 | -0.52 – 0.60 | 0.895 |
| Duration [9 s]*block order [cue–no cue]*interference [no-beat] |  | 0.71 | 0.06 – 1.35 | **0.032** |
| Duration [10 s]*block order [cue–no cue]*interference [no-beat] |  | 0.35 | -0.30 – 0.99 | 0.290 |
| Duration [1 s]*block order [cue–no cue]*interference [none] |  | -0.05 | -0.61 – 0.51 | 0.853 |
| Duration [2 s]*block order [cue–no cue]*interference [none] |  | -0.01 | -0.56 – 0.55 | 0.986 |
| Duration [3 s]*block order [cue–no cue]*interference [none] |  | -0.01 | -0.57 – 0.55 | 0.964 |
| Duration [4 s]*block order [cue–no cue]*interference [none] |  | -0.05 | -0.61 – 0.51 | 0.860 |
| Duration [5 s]*block order [cue–no cue]*interference [none] |  | -0.10 | -0.66 – 0.46 | 0.733 |
| Duration [6 s]*block order [cue–no cue]*interference [none] |  | -0.28 | -0.84 – 0.28 | 0.321 |
| Duration [7 s]*block order [cue–no cue]*interference [none] |  | -0.15 | -0.71 – 0.41 | 0.603 |
| Duration [8 s]*block order [cue–no cue]*interference [none] |  | -0.18 | -0.74 – 0.38 | 0.521 |
| Duration [9 s]*block order [cue–no cue]*interference [none] |  | 0.38 | -0.26 – 1.03 | 0.245 |
| Duration [10 s]*block order [cue–no cue]*interference [none] |  | -0.32 | -0.97 – 0.32 | 0.326 |
| Cue [yes]*duration [1 s]*block order [cue–no cue]*interference [no-beat] |  | -0.35 | -1.14 – 0.44 | 0.389 |
| Cue [yes]*duration [2 s]*block order [cue–no cue]*interference [no-beat] |  | -0.31 | -1.10 – 0.48 | 0.440 |
| Cue [yes]*duration [3 s]*block order [cue–no cue]*interference [no-beat] |  | -0.18 | -0.97 – 0.62 | 0.663 |
| Cue [yes]*duration [4 s]*block order [cue–no cue]*interference [no-beat] |  | -0.41 | -1.20 – 0.38 | 0.310 |
| Cue [yes]*duration [5 s]*block order [cue–no cue]*interference [no-beat] |  | -0.27 | -1.06 – 0.52 | 0.499 |
| Cue [yes]*duration [6 s]*block order [cue–no cue]*interference [no-beat] |  | -0.22 | -1.02 – 0.57 | 0.578 |
| Cue [yes]*duration [7 s]*block order [cue–no cue]*interference [no-beat] |  | -0.07 | -0.86 – 0.73 | 0.870 |
| Cue [yes]*duration [8 s]*block order [cue–no cue]*interference [no-beat] |  | -0.24 | -1.04 – 0.55 | 0.547 |
| Cue [yes]*duration [9 s]*block order [cue–no cue]*interference [no-beat] |  | -1.07 | -1.99 – -0.16 | 0.631 |
| Cue [yes]*duration [10 s]*block order [cue–no cue]*interference [no-beat] |  | -1.02 | -1.93 – -0.10 | 0.597 |
| Cue [yes]*duration [1 s]*block order [cue–no cue]*interference [none] |  | 0.04 | -0.75 – 0.83 | 0.923 |
| Cue [yes]*duration [2 s]*block order [cue–no cue]*interference [none] |  | 0.12 | -0.67 – 0.91 | 0.766 |
| Cue [yes]*duration [3 s]*block order [cue–no cue]*interference [none] |  | 0.16 | -0.63 – 0.95 | 0.688 |
| Cue [yes]*duration [4 s]*block order [cue–no cue]*interference [none] |  | 0.08 | -0.71 – 0.87 | 0.837 |
| Cue [yes]*duration [5 s]*block order [cue–no cue]*interference [none] |  | 0.13 | -0.66 – 0.92 | 0.746 |
| Cue [yes]*duration [6 s]*block order [cue–no cue]*interference [none] |  | 0.18 | -0.61 – 0.97 | 0.647 |
| Cue [yes]*duration [7 s]*block order [cue–no cue]*interference [none] |  | 0.07 | -0.72 – 0.86 | 0.857 |
| Cue [yes]*duration [8 s]*block order [cue–no cue]*interference [none] |  | -0.04 | -0.83 – 0.75 | 0.919 |
| Cue [yes]*duration [9 s]*block order [cue–no cue]*interference [none] |  | -0.52 | -1.43 – 0.40 | 0.269 |
| Cue [yes]*duration [10 s]*block order [cue–no cue]*interference [none] |  | -0.02 | -0.93 – 0.89 | 0.962 |
| **Random Effects** |  |  |  |  |
| σ^2^ |  | 1.06 |  |  |
| τ_00_ _subject_ |  | 0.35 |  |  |
| ICC |  | 0.25 |  |  |
| N _subject_ |  | 155 |  |  |
| Observations |  | 17763 |  |  |
| Marginal R^2^ / Conditional R^2^ |  | 0.095 / 0.318 |  |  |

**Supplementary Table C**. *Mean and variability measures per cue conditions (Cue vs. No-cue) and interval duration.*

|  |  |  | No-cue |  |  | Cue |  |  |  |  |
| --- | --- | --- | --- | --- | --- | --- | --- | --- | --- | --- |
| Interval duration (s) |  | Mean | SD | CV | Mean | SD | CV |  |  |  |
| < 0 |  | 0.311 | 0.732 | 2.353 | 0.626 | 1.269 | 2.027 |  | |  |
| 1 |  | 1.159 | 1.008 | 0.869 | 1.382 | 1.240 | 0.897 |  |  |  |
| 2 |  | 2.065 | 1.001 | 0.484 | 2.260 | 1.218 | 0.538 |  |  |  |
| 3 |  | 3.040 | 1.147 | 0.377 | 3.225 | 1.356 | 0.420 |  |  |  |
| 4 |  | 3.965 | 1.363 | 0.343 | 3.975 | 1.364 | 0.343 |  |  |  |
| 5 |  | 4.971 | 1.537 | 0.320 | 4.840 | 1.557 | 0.321 |  |  | |
| 6 |  | 5.593 | 1.690 | 0.302 | 5.639 | 1.665 | 0.295 |  |  |  |
| 7 |  | 6.441 | 1.908 | 0.296 | 6.342 | 1.753 | 0.276 |  |  |  |
| 8 |  | 7.145 | 1.963 | 0.274 | 7.132 | 1.808 | 0.253 |  |  |  |
| 9 |  | 7.637 | 2.080 | 0.272 | 7.651 | 1.886 | 0.246 |  |  |  |
| 10 |  | 8.207 | 2.009 | 0.244 | 8.368 | 1.858 | 0.222 |  |  |  |

**
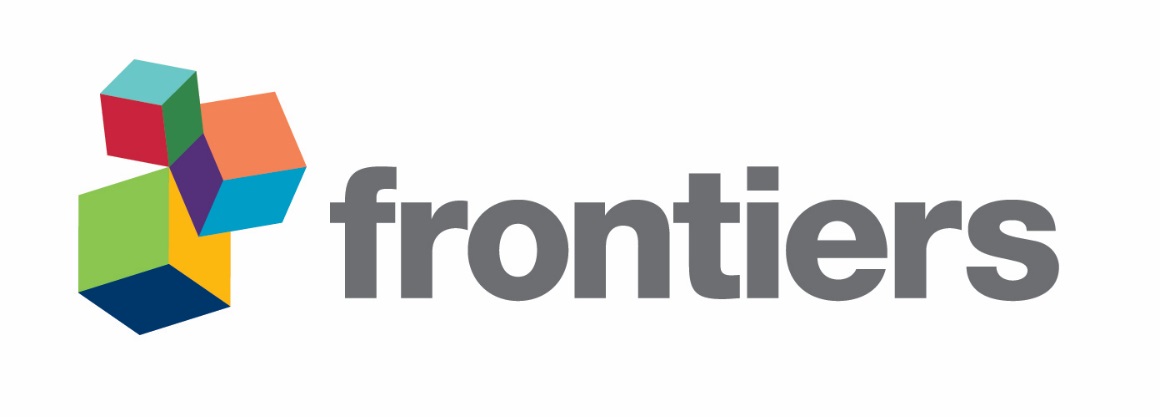
**
